# Nutritional Quality Assessment of Miscellaneous Cassava Tubers Using Principal Component Analysis and Cluster Analysis

**DOI:** 10.3390/foods13121861

**Published:** 2024-06-13

**Authors:** Lintao Chen, Rui Chen, Elsayed M. Atwa, Mahmoud Mabrouk, Huanyu Jiang, Xiangwei Mou, Xu Ma

**Affiliations:** 1 Department of Mechanical Engineering, Guangxi Normal University, Guilin 541004, China; chenlintao@mailbox.gxnu.edu.cn (L.C.);; 2 College of Biosystems Engineering and Food Science, Zhejiang University, Hangzhou 310058, China; 3 Agricultural Engineering Research Institute, Agricultural Research Center, Giza 12619, Egypt; 4 Faculty of Urban and Regional Planning, Cairo University, Giza 12613, Egypt; 5 College of Engineering, South China Agricultural University, Guangzhou 510642, China

**Keywords:** cassava planting, determination of nutritional composition, correlation analysis, standardized data processing, comprehensive evaluation

## Abstract

Cassava is a staple crop in developing countries because its starchy roots provide essential dietary carbohydrates. The aim of this research was to conduct a comprehensive inquiry and scientific evaluation of the nutritional value of cassava tubers. Eight nutritional characteristics were examined in native and imported cassava variants: starch, reduced sugar, anthocyanins, protein, dietary fiber, quinic acid, vitamin C, and dry matter content. Principal component analysis (PCA) was conducted to minimize the dimensionality of the nutritional markers. A scientific assessment technique was developed to calculate a composite score for the various cassava samples. Analysis of the data revealed noticeable variance among the samples’ nutritional indicators, suggesting varying degrees of association. Starch had a substantial positive link with lower sugar, protein, and dry matter content (*p* < 0.01). Anthocyanins and quinic acid interacted favorably (*p* < 0.05), and a positive link between protein and dry matter content was observed (*p* < 0.05); however, protein and dietary fiber interacted negatively (*p* < 0.05). The contribution rate of the top three PCA factors was over 76%, demonstrating that these factors incorporated the primary information acquired from the eight original nutritional indices, while maintaining excellent representativeness and impartiality. The experimental results showed a preliminary nutritional grade for 22 cassava tuber samples. The top five types were Guangxi Muci, Gui Cassava 4, Glutinous Rice Cassava, Huifeng 60, and Dongguan Hongwei. In the cluster analysis, the levels of similarity between the data showed that the 22 types of cassava tubers could be grouped into five categories, each with their own set of nutrients. This study promotes the directed breeding of cassava species and offers a theoretical foundation for creating and using various cassava varieties. Furthermore, this work lays the groundwork for a systematic and dependable technique for the quality assessment, comprehensive evaluation, and reasonable classification of cassava species and similar crops.

## 1. Introduction

Cassava (Manihot esculenta Crantz) is a sturdy shrub that typically grows to heights of 2–5 m. It has substantial roots that contain high amounts of carbohydrates and fiber. Propagation involves stem cuttings, specifically 13–17 cm long seed stems. After trenching, seed stems are planted horizontally or obliquely [1,2]. Beyond their rich biodiversity, cassava tuber stems and leaves can be transformed into many products, and more than 2000 variants, such as starch, alcohol, and organic chemicals, can be derived from them. Additionally, processing allows for its widespread use across the food and medicine industries, among other light industries. Cassava, a renewable biomass energy crop, is a raw material for fuel ethanol production. Breeders and molecular biologists have recently introduced high-sugar and high-starch cassava varieties adapted for post-harvest storage. However, these varieties exhibit differential nutritional composition changes. Such differences are conditioned by specific geographical influences, including the regional climate, soil fertility, and fertilization levels in growing regions [3]. Conducting a quality evaluation to select superior varieties in China’s predominant cassava-cultivating region is crucial to the cassava industry’s progress [4]. While evaluating cassava traits accurately with a single index analysis proves challenging, a comprehensive evaluation would enable a more objective and precise appraisal of cassava varieties, effectively bypassing the limitations of a single index.

PCA is an effective and popular dimension reduction and linear transformation method for distilling multiple variables into a concise set of integrated variables [5,6]. These composite indicators encapsulate most of the information present in the original variables, while remaining mutually exclusive. This process allows for the reduction of complex factors into several principal components, thus simplifying the issue and facilitating the acquisition of more scientifically reliable and efficient data. In addition, cluster analysis identifies statistical measures that gauge the degree of similarity between samples or indicators. Utilizing these statistical measures, samples with significant similarities are aggregated into classes [7]. PCA has been used for quality analysis across various agriproducts, such as potatoes, rice, and wheat. Goncalves et al. employed PCA for data dimension reduction and hierarchical clustering (HCPC) to form clusters [8]. The effectiveness of these clusters was evaluated based on coffee yield variance, thereby informing the cultivation of novel soil management methods for coffee production. Similarly, Ephzibah et al. designed a model utilizing PCA and multi-label fuzzy classifier techniques to help farmers diagnose disease categories in rice crops, enabling timely interventions [9].

Mengistu et al. employed PCA and cluster analysis to evaluate the genetic diversity of wheat [10]. Their findings were invaluable for designing and implementing new wheat breeding programs. Seid et al. leveraged PCA and clustering to select genetically diverse parents, ascertain genetic diversity and factors impacting potato genotypes, and develop high-yield, superior-quality potatoes [11]. He et al. assessed potato germplasm adaptability in Guangxi, China, using a comparative analysis integrating PCA and comprehensive systematic cluster evaluation methods [12]. Their research identified seven exceptional potato germplasms suitable for widespread application in Guangxi. Xu et al. used PCA and cluster analysis to evaluate nutrition quality across diverse rice varieties, dividing all quality indicators into five classifications [13]. Their research provided a scientific foundation for nutritional quality assessment and initiated novel concepts for the production of new functional rice products. Yu et al. selected 23 mature fruits from Huangpi germplasm resources, measured 11 species, and determined representative Huangpi quality evaluation indicators [14]. Cluster analysis was used to divide 23 Huangpi germplasms into four categories based on their quality scores. Many scholars have used PCA and cluster analysis to classify and evaluate various food crops and cassava. These studies based on PCA and cluster analysis, as well as mating techniques to develop varieties more suitable for local cultivation, are valuable for local breeding programs. Such techniques ensure higher nutritional values and economic benefits. Therefore, PCA and cluster analysis have substantial potential for applications in cassava tuber quality research. In this study, 8 key indicators were selected from 22 cassava varieties, and a comprehensive quality evaluation and classification of various cassava tuber varieties were conducted using PCA and cluster analysis.

## 2. Materials and Methodological Framework

### 2.1. Devices and Materials

Various precise instruments were employed in this study, including a precision electronic balance, a high-speed centrifuge, and a liquid chromatography–mass spectrometer. Electronic balance scales and dryers were also used at various stages of the research methodology. Regarding the test materials, this research encompassed 22 distinctive varieties of cassava roots (Guilin Academy of Agricultural Sciences, Guilin, China). The varieties were as follows: C1115, SC9, Nanzhi 199, GR 4, SC6068, Dongguan Hongwei, Glutinous Rice Cassava, Qiongzhong 1, Guangdong 1, Colombia 8H, Switzerland D32, Hainan Hongxin, Xinglong 1, Huifeng 60, Guangxi Muci, Huixian Baipi, Rongyang 1, Swiss P11, Gui Cassava 4, Shatian Bread, TMS60444, and BRA900. Each variety has its unique characteristics and nutritional profile, contributing to the variety and robustness of the quality evaluation.

### 2.2. Determination of Indicators

To determine the composition, ten uniform-sized roots were randomly selected from each variety, and a wide range of nutrients and substances, including starch, reduced sugar, anthocyanins, protein, dietary fiber, quinic acid, vitamin C, and dry matter content, were assessed using precise techniques. Regarding nutritional value, the starch content in cassava tubers is quite high. Starch and reducing sugars are the major energy sources, while anthocyanins are powerful antioxidants. Cassava also contains a certain amount of protein and can be used as a source of complementary protein. Dietary fiber is much less abundant in cassava, but cassava can still promote gastrointestinal peristalsis and reduce the time that feces stays in the body. Quinacid and vitamin C are powerful anti-inflammatory antioxidants, and vitamin C is an important substance involved in the synthesis of collagen. The dry matter contains the main nutrients of cassava, such as protein, starch, and fiber, and this content directly affects the cassava yield.

The measurement method was as follows: First, the method of determining starch in foods was followed as outlined by the national standard GB 5009.9-2016 (2016) [15]. The starch was hydrolyzed into disaccharides with amylase, followed by hydrolysis into monosaccharides after removing fats and soluble sugars. The reduced sugar measurement was then converted into starch content. Second, reduced sugar was measured using the 3,5-dinitrosalicylic acid method [16]. Reduced sugar was oxidized into sugar acid and other products under alkaline conditions. 3,5-Dinitro salicylic acid was transformed into red–brown-colored 3-amino-5-nitro salicylic acid. The substance’s absorbance was measured at 540 nm, and the proportion of reduced sugar was determined using colorimetry. Third, anthocyanins were measured using the extraction methods of Zhang Cong et al. and Wu Peng et al. [17,18]. The samples for the test were prepared with the following conditions: pH 2, 80% ethanol, and a materials-to-liquid ratio of 1:10 g/mL. Then, a portion was extracted for 40 min under 300 W ultrasonic power conditions and centrifuged at 8000 r/min, and 1 mL of its supernatant was analyzed. Fourth, protein was measured in accordance with the national standard GB 5009.5-2016 (2016) [15]. Protein in cassava was decomposed under catalytic heating conditions, and the resulting ammonia was combined with sulfuric acid to form ammonium sulfate. After alkaline distillation, the ammonia was released and absorbed with boric acid. The solution was titrated with a sulfuric or hydrochloric acid standard. The nitrogen content was computed based on the acid’s consumption and then multiplied by a conversion coefficient to determine the protein content, as shown in Equation (1).

(1) $$X=\frac{(V_{1}-V_{2})\times c\times 0.0140}{m\times V_{3}/100}\times F\times 100$$

where X is the protein content of the sample (g/100 g), V_1_ is the volume of standard titrant of sulfuric or hydrochloric acid consumed by the test solution (mL), V_2_ is the volume of sulfuric acid or hydrochloric acid standard titrant consumed by reagent blanks (mL), c is the concentration of standard titration solution of sulfuric or hydrochloric acid (mol/L), 0.0140 is the mass of the nitrogen equivalent to 1.0 mL of standard titration solution of sulfuric or hydrochloric acid (g), V_3_ is the volume of digestive solution aspirated (mL), F is the nitrogen-to-protein conversion factor, and 100 is the conversion coefficient.

Fifth, dietary fiber was determined according to the national standard GB 5009.88-2014 (2014) [19]. A dry sample was digested with α-amylase, protease, and glucosidase to remove protein and starch. Post-enzymatic hydrolysis, the solution was precipitated and filtered with ethanol, and the residue was washed with ethanol and acetone. The material was weighed upon drying to determine the total dietary fiber residue. Sixth, quinic acid was measured using the methods of Yao Gaifang et al. and Liu Wanjun et al. [20,21]. The method involved grinding 2 g of the pulp in 10 mL of deionized water, placing the sample in a water bath at 37 °C for 30 min, ultrasonic extraction for 15 min, and then centrifugation at 4 °C and 12,000 g for 15 min. In the HPLC analysis, 1 mL of supernatant was passed through a C18 SPE column and a 0.45 m Sep-Pak microporous membrane to determine the quinic acid content. Seventh, vitamin C was measured using 2,6-dichlorindiindiophol titration [22,23]. To create a homogenate, 5.0 g of cassava root was ground with 2% oxalic acid and poured into a 50 mL volumetric flask. After being rinsed several times with 2% oxalic acid and centrifuged at 9000 r/min for 10 min, 7 mL of the supernatant was titrated with a standard solution of 2,6-dichlorophinophentil until a 30 s endpoint. The consumed volume was then recorded, and the vitamin C content was calculated. Eighth, the dry matter content was determined using a standard drying method.

### 2.3. Analytical Methods

**Figure 1:**
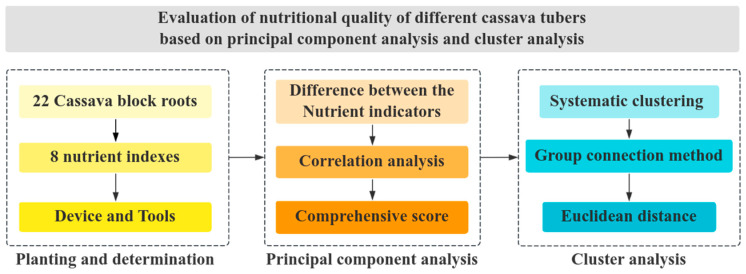
Methodological framework.

The analytical flow of this research is illustrated in Figure 1. The study included sourcing common local and imported cassava varieties to serve as test materials. A total of 22 tubers from 22 cassava varieties and eight indicators of nutritional value were selected for planting and nutrient content determination. Then, utilizing the PCA method, we calculated the differences and varying degrees of correlation among the nutritional value indicators, leading to comprehensive scoring. Ultimately, cluster analysis was employed through inter-group linkages to cluster the eight nutritional value indicators of cassava tubers, effectively serving as characteristic identifiers. Following this, we designed and evaluated a genealogical map. The data were statistically analyzed using Origin 2022 and SPSS 26.0 software. This analysis revealed a significant difference between the various groups, followed by PCA and cluster analysis. A scientific and quantitative evaluation system was established by analyzing and simplifying the nutritional value index. This system allowed for comprehensive scoring of each cassava sample.

## 3. Results and Analysis

### 3.1. Comparative Analysis

Based on the observations made in Table 1 and Table 2, the 22 cassava nutrients displayed varying traits. The anthocyanin content showed the most significant variation, with a coefficient of variation of 121.98%. This was followed by the reduced sugar content, with a coefficient of variation of 106.79%. Next were dietary fiber, vitamin C, dry matter content, quinic acid, and protein. The starch content had the slightest variation, with a relatively low coefficient of variation of 3.10%. Note that these findings display specific differences compared to previous studies [4]. This is likely due to variations in the cassava varieties used, differences in field management practices, and nuances in the ecological environment present during the experiment.

### 3.2. Correlation Analysis

**Figure 2:**
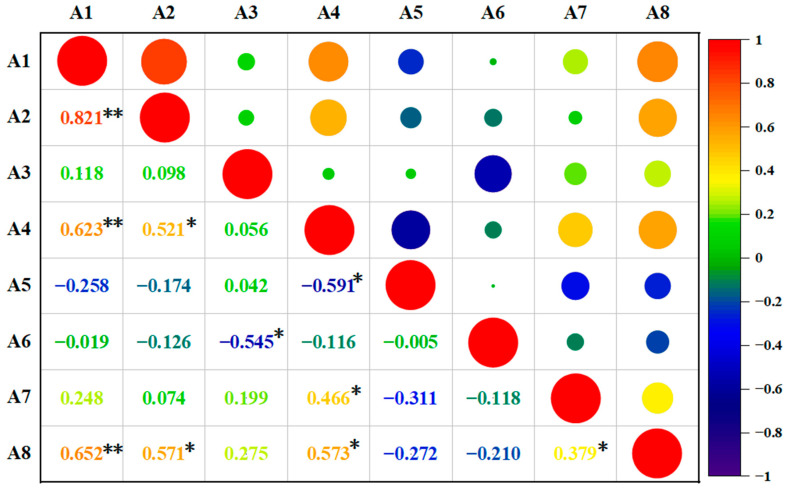
Correlation analysis of nutritional value indicators of different varieties of cassava tubers. Note: * indicates a significant correlation (*p* < 0.05); ** indicates a highly significant correlation (*p* < 0.01).

Our findings indicated significant differences in nutrient indices among the samples, as shown in Figure 2. Various degrees of correlation were also noted among these nutrient indices. A significant positive correlation was observed between starch content and reduced sugar, protein, and dry matter content (*p* < 0.01). Reducing sugars correlated significantly with protein and dry matter content (*p* < 0.05). Moreover, a positive correlation was observed between the contents of anthocyanins and quinic acid (*p* < 0.05). Protein was positively correlated with dry matter content (*p* < 0.05) but negatively correlated with dietary fiber (*p* < 0.05). Finally, vitamin C exhibited a positive correlation with dry matter content (*p* < 0.05) but a negative correlation with dietary fiber (*p* < 0.05).

### 3.3. Principal Component Analysis

#### 3.3.1. Standardized Data Processing

**Figure 3:**
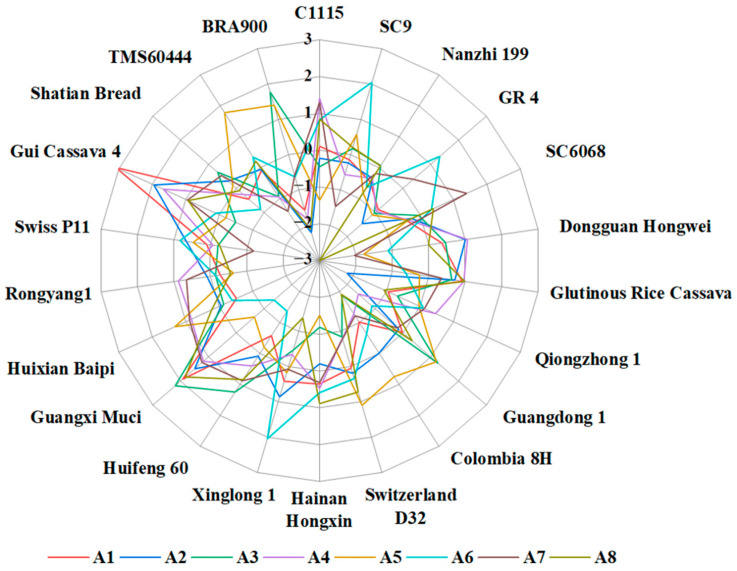
A radar plot of nutritional value indicators for different varieties of cassava tubers.

Given the varying data scales, the magnitude of each indicator differed significantly. These indicators posed a challenge, as higher numbers may amplify their respective indicators’ roles and diminish in influence at lower numerical levels. This process would not be conducive to a comprehensive evaluation analysis using Euclidean distances [24]. To tackle this issue and achieve a more balanced overall evaluation, it was necessary to normalize the nutrient composition values of the 22 assessed cassava tubers. A nonlinear normalization method was utilized in this study to standardize these values into a non-dimensional form. The outcomes of this process are displayed in Table 3 and Figure 3.

#### 3.3.2. Appropriability Test

The Kaiser–Meyer–Olkin test (KMO) and Bartlett’s test of sphericity are commonly employed in statistics to evaluate whether data are suitable for factor analysis and correlation analysis. The KMO test is used to assess the adequacy of a sample and the correlation between variables. If the KMO value is close to 1, the correlation between the variables is robust, the sample is sufficient, and the factor analysis method is suitable. The Bartlett sphericity test is used to evaluate independence between variables. A value less than 0.05 indicates a correlation between variables. In this study, the KMO measure of sampling adequacy had a value of 0.703, which exceeded the advisable threshold of 0.7. Coupled with the fact that the significance derived from Bartlett’s test of sphericity was less than 0.05, these results emphasized a robust correlation among the variables, making them suitable for PCA. Therefore, PCA could be effectively used to evaluate and interpret these data and identify the principal components or features that explained the most variance in the dataset.

#### 3.3.3. Selection of PCA

**Figure 4:**
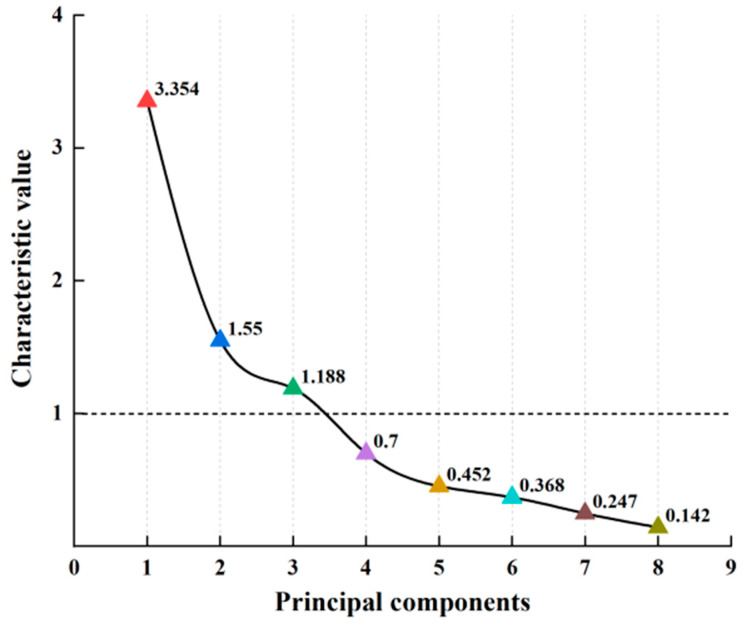
Bottom lithotripsy map of main components.

We moved forward with PCA and calculated the variance and cumulative contribution rates following the dimensionless standardization of nutrient components in the 22 cassava tubers [25]. In Table 4, the first principal component had an eigenvalue of 3.354 and contributed to 41.921% of the variance. The second principal component had an eigenvalue of 1.550, accounting for 19.369% of the variance. The third principal component, with an eigenvalue of 1.188, contributed 14.852% of the variance. By adding these rates, we found that the cumulative variance contribution rate of the first through third principal components exceeded 76.1%. As per Figure 4, there was an inflection point at the third principal component. Beyond this point, the eigenvalues fell below 1, and the trend line flattens. This suggests that the first three principal components could adequately represent the characteristic information of the eight traits across the 22 cassava tuber samples. Thus, these first three principal components could be selected as a comprehensive evaluation index for assessing the nutritional value of cassava tubers.

#### 3.3.4. Comprehensive Evaluation

**Figure 5:**
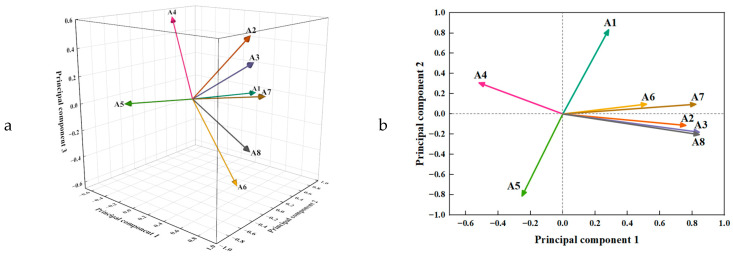

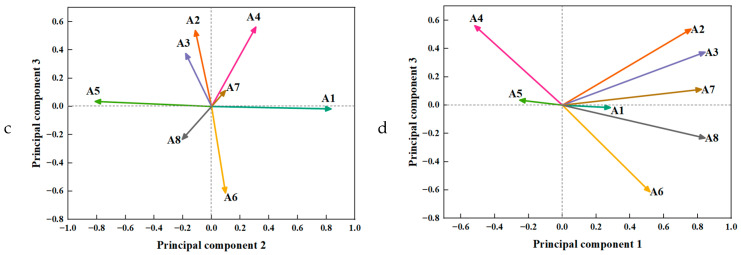
PCA diagrams: (**a**) three-dimensional load diagram; (**b**) diagram of first and second principal component load; (**c**) diagram of second and third principal component load; (**d**) diagram of first and third principal component load.

A PCA load matrix reflects each trait’s relative magnitude and the direction of its respective contribution to the principal components. In other words, it illustrates how each trait impacts the principal components, as shown in Table 5. A principal component load map, shown in Figure 5, was constructed to examine further the relationships between each principal component and the indices. These relationships are shown in diagrams for the first and second, second and third, and first and third pairs of principal component loads. In the analysis, A3, A8, A7, and A2 showed more significance in the direction of the first principal component. Similarly, A1 and A5 had significant relevance to the second principal component. A6 and A4 were noteworthy for their alignment with the third principal component direction. These observations further underlined the unique influence of different traits on the individual principal components, providing a more nuanced understanding of their contribution to the comprehensive nutritional evaluation of cassava tubers.

According to the analysis, the first principal component of cassava tubers represents traits associated with reduced sugar, anthocyanins, vitamin C, and dry matter content. On the other hand, the second principal component predominantly reflects starch and dietary fiber characteristics, while the third principal component represents protein and quinic acid. In the principal component expression, the magnitude of each factor coefficient can provide information about the extent of the factor’s contribution to that specific principal component. Notably, a load greater than 0.3 is generally considered significant in PCA. We constructed expressions for the three principal components, as shown in Equations (2)–(4), as per the eigenvectors of the correlation matrix of the nutrient components in the cassava tubers mentioned in Table 6.

(2) $$F_{1}=0.251A1+0.227A2+0.084A3+0.251A4-0.154A5-0.075A6+0.154A7+0.245A8$$

(3) $$F_{2}=-0.116A1-0.073A2+0.537A3-0.132A4+0.199A5-0.523A6+0.063A7+0.062A8$$

(4) $$F_{3}=0.316A1+0.453A2-0.014A3-0.196A4+0.473A5+0.031A6-0.515A7+0.095A8$$

Considering both the principal component coefficients and their corresponding variance contribution rates, a comprehensive evaluation could be formulated, as shown in Equation (5).The principal components and composite scores of different varieties of cassava tubers were calculated according to Equations (2)–(5) as shown in Table 7.

(5) $$Fc=0.41921F_{1}+0.19369F_{2}+0.14852F_{3}$$

**Figure 6:**
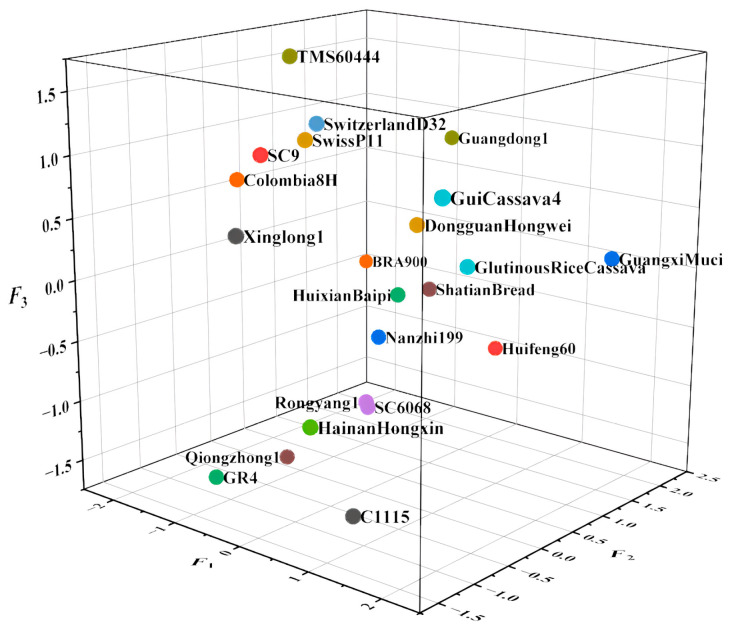
Principal component scores of cassava tuber nutrients.

**Figure 7:**
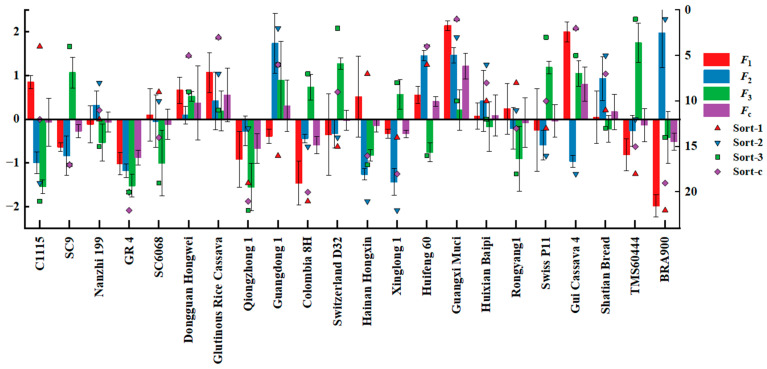
Load diagram of PCA of cassava tubers of different varieties.

As shown in Table 8, the sequence of the first five cassava types consisted of Guangxi Muci, Gui Cassava 4, Glutinous Rice Cassava, Huifeng 60, and Dongguan Hongwei. Figure 6 shows the distribution map of the principal component scores, while Figure 7 illustrates the load diagram resulting from the PCA of diverse cassava tuber varieties. Varieties with higher comprehensive scores are predominantly localized in the positive direction of F1, revealing that principal component 1 exerts substantial influence over the composite score.

### 3.4. Cluster Analysis

**Figure 8:**
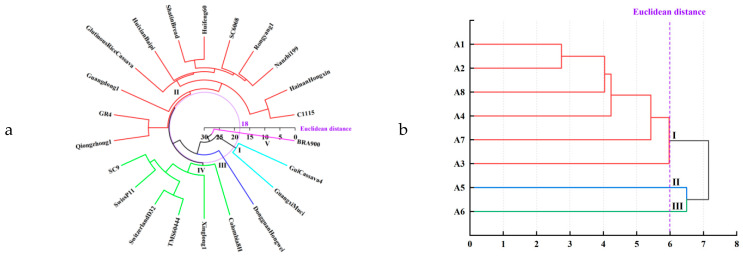
Results of systematic clustering analysis of cassava tubers: (**a**) Q-type clustering of nutritional value indicators of 22 cassava tubers; (**b**) R-type clustering of nutritional value indicators of 22 cassava tubers.

The system clustering analysis strategy can be utilized to categorize test samples into separate groups for in-depth evaluation and study. This division of samples is termed Q-type clustering, while the categorization of variables is R-type clustering. The results derived from this system are comprehensive, objective, and based on scientific principles [25]. In the experiment of this study, PCA was utilized and clustering analysis was systematically applied to organize eight nutritional value indicators from different strains of cassava tuber into R-type clusters and 22 cassava tuber strains into Q-type clusters. Figure 8 depicts the results of the clustering process. With a Euclidean distance of 18, 22 cassava strains were classified into five main categories. Figure 8 shows that C1115, Hainan Hongxin, Nanzhi 199, and Rongyang 1, among others, were assigned to one category; SC9, Swiss P11, Switzerland D32, and TMS60444 were clustered in another category; Guangxi Muci and Gui Cassava 4 belonged to a separate category; and Dongguan Hongwei and BRA900 formed individual categories.

Table 8 outlines the clustering analysis of nutritional value indicators from the 22 cassava types. Category I comprises Guangxi Muci and Gui Cassava 4. These varieties are locally sourced, demonstrate favorable nutritional value, and possess high starch content, reducing sugars, dry matter content, and other nutrients, rendering them excellent candidates for selection. Category II includes Glutinous Rice Cassava, Huifeng 60, and 12 varieties, mainly from localities in Southern China. They offer moderate nutritional value, a balanced protein content, and a subset of varieties with elevated individual nutrient content—qualities that lend them significant potential for selection. Category III contains Dongguan Hongwei, which exhibits exceptional protein content, making it a suitable choice. Six strains, including SwissP11 and Switzerland D32, are included in Category IV. Despite the introduction of varieties, they have commendable fiber content. Regardless of these limitations, some varieties have a comparatively low content of certain nutrients but maintain a high selection value. Finally, category V, represented by BRA900, has a higher anthocyanin content but lower levels of individual nutrients. As a result, selection from this class of cassava would primarily depend on specific production circumstances.

## 4. Discussion

At present, multivariate statistical methods, specifically PCA, are widely used to assess the agronomic traits and nutritional quality of crops. In addition, there is cluster analysis, which can divide an unknown category of crops into several clusters to further explore the relationship between the species. In this study, the results from the PCA, which classified the eight nutrients into three primary components, and the cluster analysis, which grouped the 22 cassava root tubers into five different categories and the eight nutrients into three categories, significantly aided the comprehensive evaluation process of cassava quality. Five superior varieties were identified: Guangxi Muci, Gui Cassava 4, Glutinous Rice Cassava, Huifeng 60, and Dongguan Hongwei.

Plant growth, development, and physiological characteristics, such as those of cassava, are influenced by the plant’s genetic material and environmental conditions like temperature, light, and water. In various regions, local climate and cultivation methods significantly influence cassava’s nutrient content. During the cassava planting period in Guangxi, limited sunlight and excessive rainfall affect some imported varieties’ yield and nutritional value, leading to reduced quality of some varieties. On the other hand, large-scale discretization was performed after excluding the error of the experimental method. The reasons for this discretization included differences in harvest time and sample preservation time.

The 22 kinds of cassava in this study were comprehensively ranked according to the nutritional value of their tubers. In addition to its nutritional value, cassava can be economically beneficial. In practical applications, different kinds of cassava have varying advantages, including having edible, feeding, industrial, and medicinal value. In addition, the leaves of some varieties have high nutritional value. At present, cassava is widely used in many fields, and the development of production in poor mountainous areas could increase farmers’ economic income, reduce the feed burden of the livestock and poultry industries, and provide raw materials for enterprises for processing and production.

## 5. Conclusions

The nutritional value indicators of the cassava root samples showed significant differences and varying degrees of correlation. In the correlation analysis, there were positive or negative correlations between multiple nutrients. The PCA extracted three primary factors with a cumulative contribution rate exceeding 76%. The first was closely related to reducing sugars, anthocyanins, vitamin C, and dry matter content; the second was mainly linked to starch and dietary fiber; and the third was primarily associated with protein and quinic acid. Cluster analysis grouped 22 cassava root samples into 5 categories and the 8 nutrients into 3 categories. Each category displayed noticeable differences in nutrient composition and Euclidean distance, suggesting unique nutritional profiles. In conclusion, cassava roots hold excellent nutritional value and excellent potential for various utilizations. However, particular applications might vary significantly across cassava varieties, due to the geographical environment and other factors. The findings in this study provide a solid theoretical basis for the targeted breeding of different cassava species and the comprehensive use and productive development of each variety. In future studies, the cassava tuber sample size will be expanded, a more comprehensive evaluation of different uses will be conducted, and an additional evaluation analysis of cassava leaves will be considered. This could facilitate the development of cassava products in a more targeted manner, depending on classification characteristics.

## Figures and Tables

**Table 1:** Results of nutritional value determination of different cassava tuber varieties.

Breed	Character							
	A1/(g·kg^−1^)	A2/(mg·kg^−1^)	A3/ (mg·kg^−1^)	A4/(g·kg^−1^)	A5/(g·100 g^−1^)	A6/(mg·g^−1^)	A7/(mg·100 g^−1^)	A8/ %
C1115	680	36.25	196.49	8.31	1.4	0.24	41.62	19.61
SC9	676	36.13	276.81	5.82	2.17	0.29	24.1	18.67
Nanzhi 199	671	35.31	263.24	6.09	1.62	0.18	32.21	18.46
GR 4	664	26.49	120.43	5.32	1.5	0.26	35.76	16.81
SC6068	672	36.02	249.45	6.09	1.8	0.22	42.23	18.96
Dongguan Hongwei	684	45.81	312.92	7.87	1.23	0.16	20.45	18.36
Glutinous Rice Cassava	693	43.57	335.46	7.75	1.84	0.18	35.6	19.82
Qiongzhong 1	663	20.95	167.44	7.12	1.93	0.21	34.25	16.79
Guangdong 1	678	36.76	416.33	4.5	2.42	0.16	32.12	18.86
Colombia 8H	662	37.88	12.67	4.92	2.24	0.18	25.68	15.56
Switzerland D32	679	39.46	146.32	5.53	2.38	0.22	28.44	19.43
Hainan Hongxin	684	36.45	101.53	7.13	1.34	0.23	35.38	19.68
Xinglong 1	685	44.69	194.16	6.1	2.01	0.29	33.88	16.34
Huifeng 60	669	38.65	414.62	7.04	1.86	0.15	38.98	19.61
Guangxi Muci	709	49.62	536.32	8.02	1.68	0.15	41.17	21.06
Huixian Baipi	670	37.55	237.04	7.58	2.47	0.19	39.01	18.71
Rongyang1	674	38.68	235.96	7.64	1.69	0.2	37.53	17.56
Swiss P11	680	43.13	227.91	6.44	2.13	0.24	25.85	17.98
Gui Cassava 4	728	53.24	190.98	8.64	1.86	0.21	39.43	19.73
Shatian Bread	671	40.29	340.29	6.2	1.99	0.17	36.73	18.21
TMS60444	677	37.56	133.74	5.34	2.65	0.22	24.48	18.68
BRA900	653	20.71	482.57	4.23	2.5	0.18	29.67	15.26

Note: A1 is starch; A2 is reduced sugar; A3 is anthocyanins; A4 is protein; A5 is dietary fiber; A6 is quinic acid; A7 is vitamin C; and A8 is dry matter content.

**Table 2:** Differential analysis of 22 different nutrient components of cassava tubers.

Item	Minimum	Maximum	Average	Standard Deviations	Coefficient of Variation/%
A1/(g·kg^−1^)	653.00	728.00	685.2346	21.2674	3.10
A2/(mg·kg^−1^)	2.71	53.24	14.2679	15.2364	106.79
A3/(mg·kg^−1^)	12.67	1198.32	242.3659	295.6314	121.98
A4/(g·kg^−1^)	4.23	8.64	7.1265	1.2697	17.82
A5/(g·100 g^−1^)	1.21	2.65	1.6984	1.3659	80.42
A6/(mg·g^−1^)	0.14	0.29	0.2059	0.04102	19.92
A7/(mg·100 g^−1^)	20.60	42.30	35	26.3658	75.33
A8/%	15.26	21.06	6.2277	1.2685	20.37

**Table 3:** Data standardization.

Breed	Character							
	A1	A2	A3	A4	A5	A6	A7	A8
C1115	0.10686	−0.21925	−0.44885	1.40851	−1.35883	0.83112	1.30117	0.84253
SC9	−0.14192	−0.23461	0.17571	−0.56283	0.57388	2.05010	−1.46860	0.20360
Nanzhi 199	−0.45290	−0.33952	0.07020	−0.34907	−0.80663	−0.63165	−0.18648	0.06086
GR 4	−0.88827	−1.46802	−1.04028	−0.95868	−1.10783	1.31871	0.37475	−10.06066
SC6068	−0.39070	−0.24868	−0.03703	−0.34907	−0.35482	0.34353	1.39760	0.40072
Dongguan Hongwei	0.35565	1.00392	0.45650	1.06016	−1.78553	−1.11925	−2.04564	−0.00711
Glutinous Rice Cassava	0.91541	0.71732	0.63177	0.96516	−0.25442	−0.63165	0.34945	0.98527
Qiongzhong 1	−0.95047	−2.17684	−0.67474	0.46639	−0.02852	0.09973	0.13603	−1.07425
Guangdong 1	−0.01753	−0.15400	1.26061	−1.60788	1.20138	−1.11925	−0.20070	0.33275
Colombia 8H	−1.01266	−0.01070	−1.87822	−1.27536	0.74958	−0.63165	−1.21882	−1.91029
Switzerland D32	0.04467	0.19146	−0.83896	−0.79242	1.10098	0.34353	−0.78248	0.72018
Hainan Hongxin	0.35565	−0.19367	−1.18725	0.47430	−1.50943	0.58733	0.31467	0.89011
Xinglong 1	0.41784	0.86062	−0.46696	−0.34115	0.17228	2.05010	0.07754	−1.38012
Huifeng 60	−0.57729	0.08782	1.24731	0.40305	−0.20422	−1.36304	0.88381	0.84253
Guangxi Muci	1.92299	1.49140	2.19364	1.17892	−0.65602	−1.36304	1.23003	1.82811
Huixian Baipi	−0.51510	−0.05292	−0.13353	0.83057	1.32688	−0.38786	0.88855	0.23079
Rongyang1	−0.26631	0.09166	−0.14193	0.87807	−0.63092	−0.14406	0.65457	−0.55087
Swiss P11	0.10686	0.66102	−0.20453	−0.07197	0.47348	0.83112	−1.19194	−0.26540
Gui Cassava 4	3.09227	1.95456	−0.49169	1.66978	−0.20422	0.09973	0.95495	0.92410
Shatian Bread	−0.45290	0.29765	0.66933	−0.26198	0.12208	−0.87545	0.52810	−0.10906
TMS60444	−0.07972	−0.05164	−0.93679	−0.94285	1.77868	0.34353	−1.40853	0.21040
BRA900	−1.57243	−2.20755	1.77569	−1.82164	1.40218	−0.63165	−0.58803	−2.11420

**Table 4:** Variance contribution rate from the PCA of cassava tubers.

Ingredient	Initial Eigenvalue			Extracted Sum of Load Squares		
	Total	Variance %	Accumulation %	Total	Variance %	Accumulation %
1	3.354	41.921	41.921	3.354	41.921	41.921
2	1.550	19.369	61.290	1.550	19.369	61.290
3	1.188	14.852	76.142	1.188	14.852	76.142
4	0.700	8.748	84.891			
5	0.452	5.653	90.544			
6	0.368	4.601	95.145			
7	0.247	3.082	98.228			
8	0.142	1.772	100.000			

**Table 5:** Principal component load matrix of the cassava tuber nutrients.

Item	Principal Component		
	1	2	3
A3	0.843	−0.180	0.375
A8	0.843	−0.204	−0.232
A7	0.822	0.096	0.112
A2	0.760	−0.113	0.538
A1	0.283	0.832	−0.017
A5	−0.252	−0.811	0.036
A6	0.517	0.097	−0.612
A4	−0.518	0.308	0.562

**Table 6:** Eigenvectors of the nutrient composition correlation matrix of the cassava tubers.

Item	Principal Component		
	1	2	3
A3	0.251	−0.116	0.316
A8	0.227	−0.073	0.453
A7	0.084	0.537	−0.014
A2	0.251	−0.132	−0.196
A1	−0.154	0.199	0.473
A5	−0.075	−0.523	0.031
A6	0.154	0.063	−0.515
A4	0.245	0.062	0.095

**Table 7:** Principal component and comprehensive score of different varieties of cassava tubers.

Breed	F_1_	Sort-1	F_2_	Sort-2	F_3_	Sort-3	Comprehensive Score (F_c_)	Sort-c
C1115	0.84661057	4	−0.99421864	19	−1.54236062	21	−0.066733991	12
SC9	−0.6338065	17	−0.8356597	17	1.06739903	4	−0.269026846	17
Nanzhi 199	−0.11468116	12	0.32295466	8	−0.52878228	15	−0.064057145	11
GR 4	−1.01465614	20	−1.17417499	20	−1.52012371	20	−0.878548728	22
SC6068	0.09704118	9	−0.04771496	10	−1.00605313	19	−0.117980288	14
Dongguan Hongwei	0.66374901	5	0.09138896	9	0.52655551	9	0.374155374	5
Glutinous Rice Cassava	1.06968427	3	0.41613291	7	0.18991147	11	0.557228778	3
Qiongzhong 1	−0.91765522	19	−0.2706029	13	−1.55093065	22	−0.667447541	21
Guangdong 1	−0.38749749	16	1.73489198	2	0.8907222	6	0.305878466	6
Colombia 8H	−1.4582733	21	−0.43771123	15	0.73260292	7	−0.587296853	20
Switzerland D32	−0.35406996	15	−0.33029663	14	1.27071413	2	−0.02367836	9
Hainan Hongxin	0.51956396	7	−1.25981747	21	−0.82493617	17	−0.148727158	16
Xinglong 1	−0.33109157	14	−1.43562142	22	0.56929818	8	−0.332310244	18
Huifeng 60	0.55717941	6	1.45730468	4	−0.75307342	16	0.40399406	4
Guangxi Muci	2.14195962	1	1.46358485	3	0.20914306	10	1.212474569	1
Huixian Baipi	0.07408384	10	0.41946144	6	−0.16775428	12	0.087387307	8
Rongyang1	0.23624327	8	−0.2110466	11	−0.9050739	18	−0.07626365	13
Swiss P11	−0.24220327	13	−0.59298305	16	1.18853622	3	−0.03986752	10
Gui Cassava 4	1.99509464	2	−0.96117721	18	1.0446656	5	0.805346945	2
Shatian Bread	0.04582152	11	0.93348237	5	−0.2180308	13	0.167633105	7
TMS60444	−0.8121248	18	−0.26698425	12	1.74667524	1	−0.13274681	15
BRA900	−1.98096505	22	1.97881552	1	−0.41908981	14	−0.509406799	19

Note: *F*_1_–*F*_3_ represents the scores of the main components of the different varieties of cassava tubers.

**Table 8:** Clustering analysis of nutritional value indicators for 22 types of cassava tubers.

Item	Mean Value				
	Category I (2)	Category II (12)	Category III (1)	Category IV (6)	Category V (1)
A1/(g·kg^−1^)	718.60	674.08	684.00	676.50	653.00
A2/(mg·kg^−1^)	51.43	35.58	45.81	39.81	20.71
A3/(mg·kg^−1^)	363.65	256.52	312.92	165.27	482.57
A4/(g·kg^−1^)	8.33	6.73	7.87	5.69	4.23
A5/(g·100 g^−1^)	1.77	1.82	1.23	2.26	2.50
A6/(mg·g^−1^)	0.18	0.20	0.16	0.24	0.18
A7/(mg·100 g^−1^)	40.30	36.79	20.45	27.07	29.67
A8/%	20.39	18.59	18.36	17.78	15.26

Note: A1 is starch; A2 is reducing sugars; A3 is anthocyanins; A4 is protein; A5 is dietary fiber; A6 is quinic acid; A7 is vitamin C; and A8 is dry matter content.

## Data Availability

The original contributions presented in the study are included in the article, further inquiries can be directed to the corresponding author.
